# Proton-Coupled Chromophore and Protein Structural Changes Control Phytochrome Activation

**DOI:** 10.1021/acs.biochem.5c00713

**Published:** 2026-02-13

**Authors:** Galaan Merga, Maximilian Große, Anastasia Kraskov, Francisco Velazquez Escobar, Norbert Michael, Manal Ebrahim, Luisa Sauthof, Patrick Scheerer, Franz Bartl, Peter Hildebrandt

**Affiliations:** † 9373 Humboldt- Universität Zu Berlin, Institut für Biologie, Biophysikalische Chemie, Invalidenstr 42, Berlin D-10115, Germany; ‡ 26524 Technische Universität Berlin, Institut für Chemie, Sekr. PC14, Straße des 17. Juni 135, Berlin D-10623, Germany; § Charité − Universitätsmedizin Berlin, Corporate Member of Freie Universität Berlin and Humboldt-Universität Zu Berlin, Institute of Medical Physics and Biophysics, Group Structural Biology of Cellular Signaling, Charitéplatz 1, Berlin D-10117, Germany

## Abstract

Phytochromes are sensory photoreceptors in eukaryotes and prokaryotes that control physiological processes. In prototypical phytochromes, photoisomerization of the methine-bridged tetrapyrrole of the Pr state is the first step in (de)­activating the photoreceptor. The underlying reaction sequence runs through a series of intermediate states. Among them, the Meta-Rc state plays a critical role since it precedes the formation of the Pfr state, which is linked to the functional secondary structure transition of the tongue, a phytochrome-specific peptide segment. In this work, we have studied the structure and reactions of Meta-Rc of the bacterial phytochrome Agp1 (*Agrobacterium fabrum*) by IR difference and resonance Raman spectroscopy. It is shown that the formation of Meta-Rc is associated with the enolization of the terminal ring D and the deprotonation of ring B or C, whereas reprotonation of the chromophore occurs with the decay of Meta-Rc. Proton migration represents the essential trigger for the secondary structure transition of the tongue since the β-sheet and α-helix structures can be interconverted by changing the pH. The pH-dependent conformational equilibrium is observed in Meta-Rc at 250 K and in Pfr at 290 K, albeit with different p*K*_A_ values. The results show that the secondary structure transition is induced by chromophore-linked proton transfer steps rather than by conformational relaxations of the chromophore itself. In view of previous findings on the proton dependence of the reverse process in bathy phytochromes, we conclude that intramolecular proton transfer is an indispensable prerequisite for the secondary structure transition in phytochromes in general.

## Graphical abstract

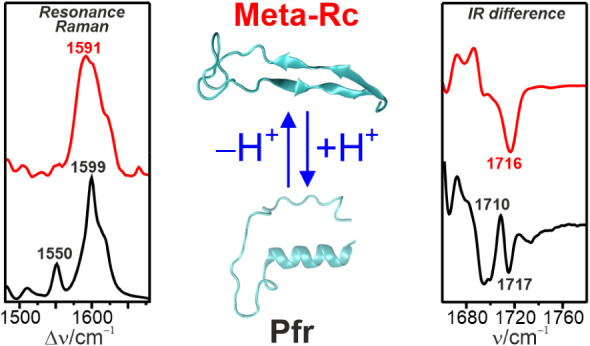

## Introduction

Phytochromes are sensory photoreceptor proteins, which control photomorphogenic processes in plants.^1–3^ They are also found in fungi and bacteria, but much less is known about their functions in these organisms.^4–7^ Despite the quite diverse cellular hosts, the various members of the phytochrome family share common structural and mechanistic properties.^3,8^ Canonical phytochromes are typically composed of two modules. The photosensory core module (PCM) is composed of PAS (Period/ARNT/Sim), GAF (cGMP-Phosphodiesterase/adenylate cyclase/FhlA), and a phytochrome-specific PHY domain. The PCM harbors a linear methine-bridged tetrapyrrole chromophore that, upon light absorption, switches between two parent states, denoted as Pr and Pfr (Figure 1).^2,9,10^

**Figure 1:**
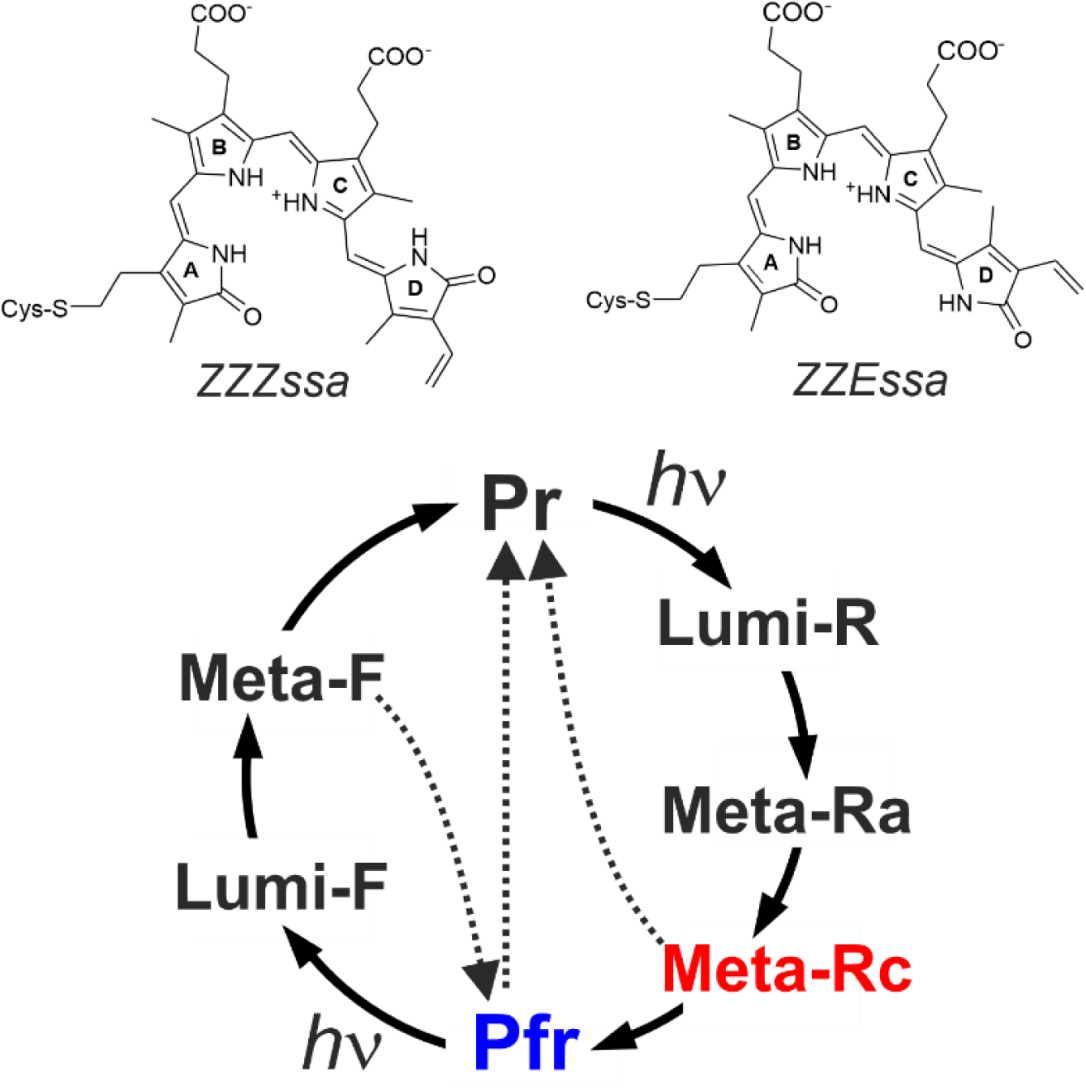
Structural formula of the biliverdin chromophore in Pr (*ZZZssa*, top left) and Pfr (*ZZEssa*, top right) and a simplified presentation of the photocycle (bottom). Pr and Pfr are the stable dark states in prototypical (e.g., Agp1) and bathy phytochromes (e.g., Agp2), respectively. The photochemical reaction steps are marked by “*h*ν”. The dotted arrows indicate thermal shortcut reactions to the initial parent states. The critical states that are in the focus of the present work are highlighted in red (Meta-Rc) and blue (Pfr).

Despite small structural variations of the chromophore, the basic reaction mechanism is very similar in all phytochromes.^11^ It starts with the photoisomerization at the methine bridge between rings C and D (C–D methine bridge) of the chromophore, which adopts *ZZZssa* and *ZZEssa* configurations in Pr and Pfr, respectively. This step is followed by a series of hierarchical conformational changes that range from structural adaptations in the chromophore-binding pocket up to the secondary structure transition of the tongue,^12^ a peptide segment in the PHY domain. The latter conformational transition triggers further protein structure changes on the tertiary and quaternary structure levels.^12–15^ These structural changes extend to the output module, typically carrying a histidine kinase, to switch its activity status between “inactive” and “active”.^16,17^

From a biophysical point of view, phytochrome is an instructive example of how structural changes are transduced over different time and length scales. A broad range of physical techniques have been employed to address this issue, including cryo- and time-resolved protein crystallography,^12,18–29^ NMR spectroscopy,^30–32^ and various optical spectroscopies, including ultrafast absorption,^33,34^ IR,^35–38^ and resonance Raman (RR) spectroscopy.^39,40^ However, despite extensive efforts and substantial progress in elucidating the structures and structural changes of phytochromes, the mechanisms that rule the cascade of structural changes are far from being fully understood.

The present study is a contribution to understanding the coupling between chromophore structural changes in the chromophore binding pocket and protein conformational transitions of the tongue. This issue has been previously addressed in mutational studies that identified critical amino acids for linking chromophore and protein structural changes.^41–43^ On the scale of the photoinduced reaction sequence, coupling of chromophore and protein structural changes occurs with the formation and decay of the Meta intermediates (Figure 1).^11^ The critical role of the Meta states also roots in their function as branching points of the reaction cascades.^44,45^ Meta-Rc in prototypical phytochromes and Meta-F in bathy phytochromes each open two reaction channels. One pathway (Meta-Rc→Pfr and Meta-F→Pr) leads to the respective final product, concomitant with the restructuring of the tongue, whereas the other pathway (Meta-Rc→Pr and Meta-F→Pfr) is unproductive and returns to the initial state.

In this work, we studied the Pr→Pfr photoconversion of the prototypical phytochrome Agp1 from *Agrobacterium fabrum*,^7^ using IR difference and RR spectroscopy, which selectively probes photoinduced structural changes of phytochromes and the vibrational modes of the chromophore, respectively.^10,46,47^ The study builds upon previous vibrational spectroscopic investigations,^11,47–49^ but the focus is now laid on the structure of Meta-Rc and its reaction to Pfr. We employed the cryotrapping technique, in which the photoconversion is initiated at a specific temperature. This temperature is chosen such that the sequence of thermal reactions is blocked by the formation of the target intermediate. The present investigations show that proton translocations perturb the structure and electrostatics of the chromophore and its immediate environment, which in turn induces restructuring of the tongue.

## Materials and Methods

### Protein Expression and Purification

Full-length Agp1 was expressed and purified as described previously.^50^ For selected measurements, the apoprotein was assembled with BV deuterated at the A–B and C–D methine bridges and the vinyl substituent of ring D (Toronto Research Chemicals). For spectroscopic experiments, dark-adapted protein was concentrated to ca. 1 mM for RR and IR difference spectroscopy. Between pH 7 and 9, the solution was buffered with Tris. For pH values outside this range, the buffer was exchanged using an Amicon Ultra centrifugal filter by washing the sample five times with the respective buffer (preadjusted to the target pH using HCl or NaOH). A pH electrode was used to verify the pH of the sample (measured at 20 °C) before and after buffer exchange. Following H/D exchange, the pD was corrected by 0.4 units.^51^ Due to the limited volume and high protein concentration, the estimated uncertainty of the pH measurement is ±0.1 pH units.

### IR Difference Spectroscopy

For IR measurements, the protein samples were placed in sample holders with a 6 μm PTFE spacer. In cryogenic IR experiments, the sample was cooled to the desired temperature with an OptistatTN cryostat (Oxford Instruments). The spectrum was recorded using a Bruker IFS66v/s spectrometer (2 cm^–1^ spectral resolution at a time resolution of ca. 120 ms) equipped with a mercury cadmium telluride (MCT) detector (J15D series, EG&G Judson). The light-induced spectrum was recorded during irradiation with λ = 685 nm for ca. 60 s, corresponding to a photon flux of ca. 3·10^20^ photons/(m^2^·s). The difference spectra calculated from the single-channel spectra before and after irradiation were preprocessed with the OPUS 7.5 software package (Bruker Optics, Karlsruhe, Germany).

### Resonance Raman Spectroscopy

RR measurements were performed using either a Bruker Fourier-transform Raman spectrometer RFS 100/S or a Bruker Fourier-transform MultiRAM spectrometer with a Ramanscope III. Both spectrometers were equipped with an Nd:YAG continuous-wave (cw) laser for 1064 nm excitation (line width 1 cm^–1^) (Bruker, Karlsruhe, Germany) and a nitrogen-cooled cryostat from Resultec (Linkam). All spectra of the samples in frozen solution were recorded at ca. 80 K with a laser power of 680 mW at the sample and an accumulation time of typically 1 h. Potential laser-induced damage to the phytochrome samples could be ruled out since comparison of RR spectra before and after a series of measurements did not reveal any changes. For photoconversion, the protein sample was brought to the required temperature and illuminated with a 670 nm laser diode for 5 min (photon flux ca. 1·10^21^ photons/(m^2^·s)). In some cases, the illumination time was adjusted to achieve full conversion. After that, the sample was cooled to 80 K for RR measurement. Residual contributions from the nonphotoconverted state or other intermediates were removed by manually weighted spectra subtraction.^52,53^

## Results

The various intermediate states of the Pr→Pfr photoconversion of Agp1 were cryotrapped at appropriate temperatures and characterized by resonance Raman (RR) and IR difference spectroscopy, which provide information about the chromophore structure and protein structural changes, respectively. We focus on the RR and IR bands that are relevant to the present study. Comprehensive vibrational assignments were published elsewhere.^11,40,48,49,52,54–56^ In both types of spectroscopic experiments, we used approximately the same trapping temperatures and, as far as possible, solution conditions to ensure probing the same phytochrome states. The temperatures for trapping Lumi-R, Meta-Ra, and Meta-Rc were ca. 130, 210, and 250 K, respectively.^10,11,52,53^

### Resonance Raman Spectra

The RR spectra of phytochromes can be divided into two regions, which are of special interest for the structural analysis of the chromophore (Figure 2).^10^ The region between 650 and 900 cm^–1^ includes vibrational modes with strong contributions from the hydrogen out-of-plane coordinates (HOOP) of the methine bridges. These modes gain RR activity upon deviations from a planar methine bridge geometry. Here the HOOP mode of the C–D methine bridge between 790 and 840 cm^–1^ is particularly important since it indicates distortions of the C–D isomerization site.^54^

**Figure 2:**
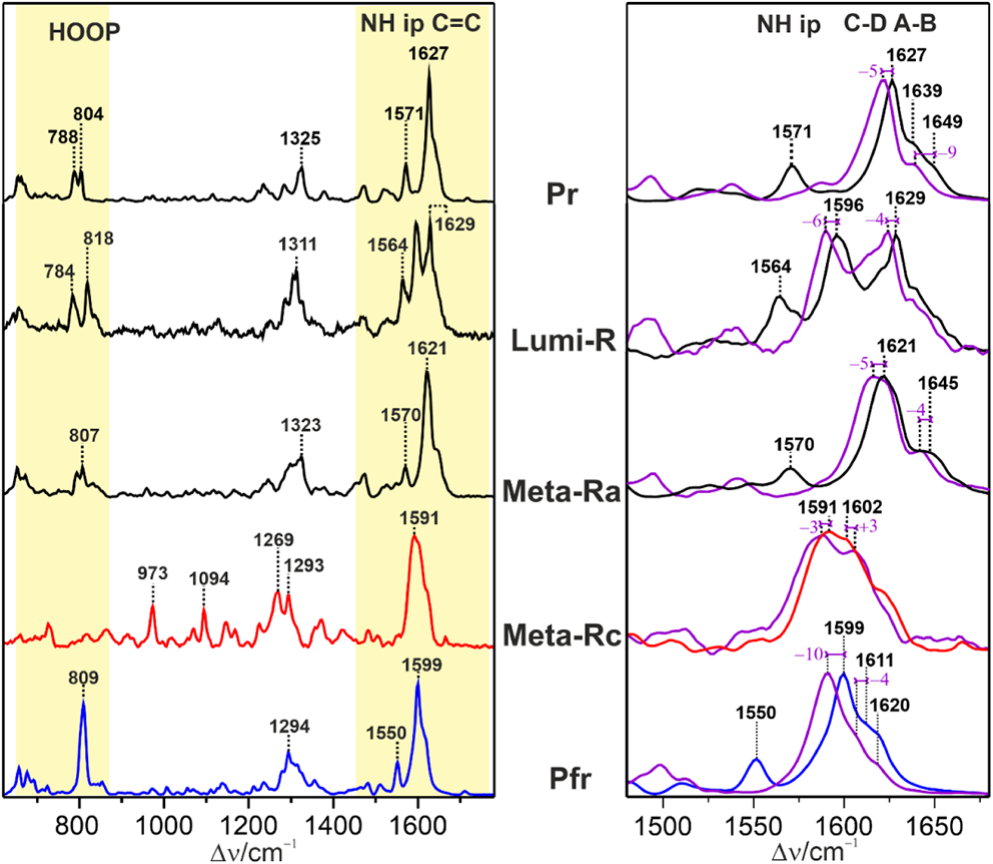
RR spectra of the various states of Pr→Pfr photoconversion of Agp1, measured at 80 K with 1064 nm excitation. The intermediates Lumi-R, Meta-Ra, and Meta-Rc (red trace) were trapped at 130, 210, and 250 K, respectively. Pfr (blue trace) was generated at 290 K. The spectra were measured at pH 7.8 in H_2_O, except for the violet traces (right panel) that refer to measurements in D_2_O. The HOOP and C=C stretching regions are highlighted in yellow (left panel), and the C=C stretching region is shown in a close-up view on the right panel.

Thus, the intense band at 818 cm^–1^ points to a twisted C–D geometry in Lumi-R,^52^ whereas upon structural relaxation in Meta-Ra and Meta-Rc, the intensity is largely reduced. However, one of the most characteristic features of Pfr is the drastic intensity increase of this mode at 809 cm^–1^. Interestingly, this peak is composed of two closely spaced bands, reflecting two substates of Pfr.^54^

The spectral region between 1500 and 1660 cm^–1^ is diagnostic of the protonation state and the overall geometry of the chromophore. The band of medium intensity between 1550 and 1575 cm^–1^ originates from the in-phase N–H in-plane bending (N–H ip) of the ring B and C N–H groups.^57^ This mode is missing in deprotonated tetrapyrroles.^10^ As a rather pure mode, it shifts down to 1050–1080 cm^–1^ upon H/D exchange.^11^ The remaining bands above 1550 cm^–1^ are assigned to stretching modes of the three methine bridges and the C=C stretching of ring D. The vibrational assignments have been discussed previously in more detail.^10,11,54,58^

Here we emphasize the stretching mode of the C–D methine bridge (C–D stretching), which gives rise to the strongest RR band of protonated tetrapyrroles. On the left and right sides of this band, shoulders of weaker intensity originate from the B–C and A–B stretching modes, respectively. In Agp1, the position of the C–D stretching varies from 1627 cm^–1^ in Pr via 1621 cm^–1^ in Meta-Ra to 1599 cm^–1^ in Pfr. In Lumi-R, the twisted geometry causes a redistribution of the vibrational coordinates, leading to two comparably intense bands at 1596 and 1629 cm^–1^, which presumably include contributions from all three methine bridge stretchings. The methine bridge stretching modes of protonated tetrapyrroles exhibit distinct H/D isotope downshifts of typically 5–10 cm^–1^ due to the admixture of the adjacent N–H ip coordinates. Considering these spectral markers, Meta-Rc is a special case since the N–H ip mode is missing and the C=C stretching region is dominated by the overlap of several broad bands with little H/D sensitivity. These findings demonstrate the lack of a protonated nitrogen on ring B or C, or both.^11,48^

### IR Difference Spectra

The IR difference spectra of the Pr→Pfr photoconversion of Agp1 reflect structural changes with respect to Pr of both the protein and the chromophore (Figure 3).^47^

**Figure 3:**
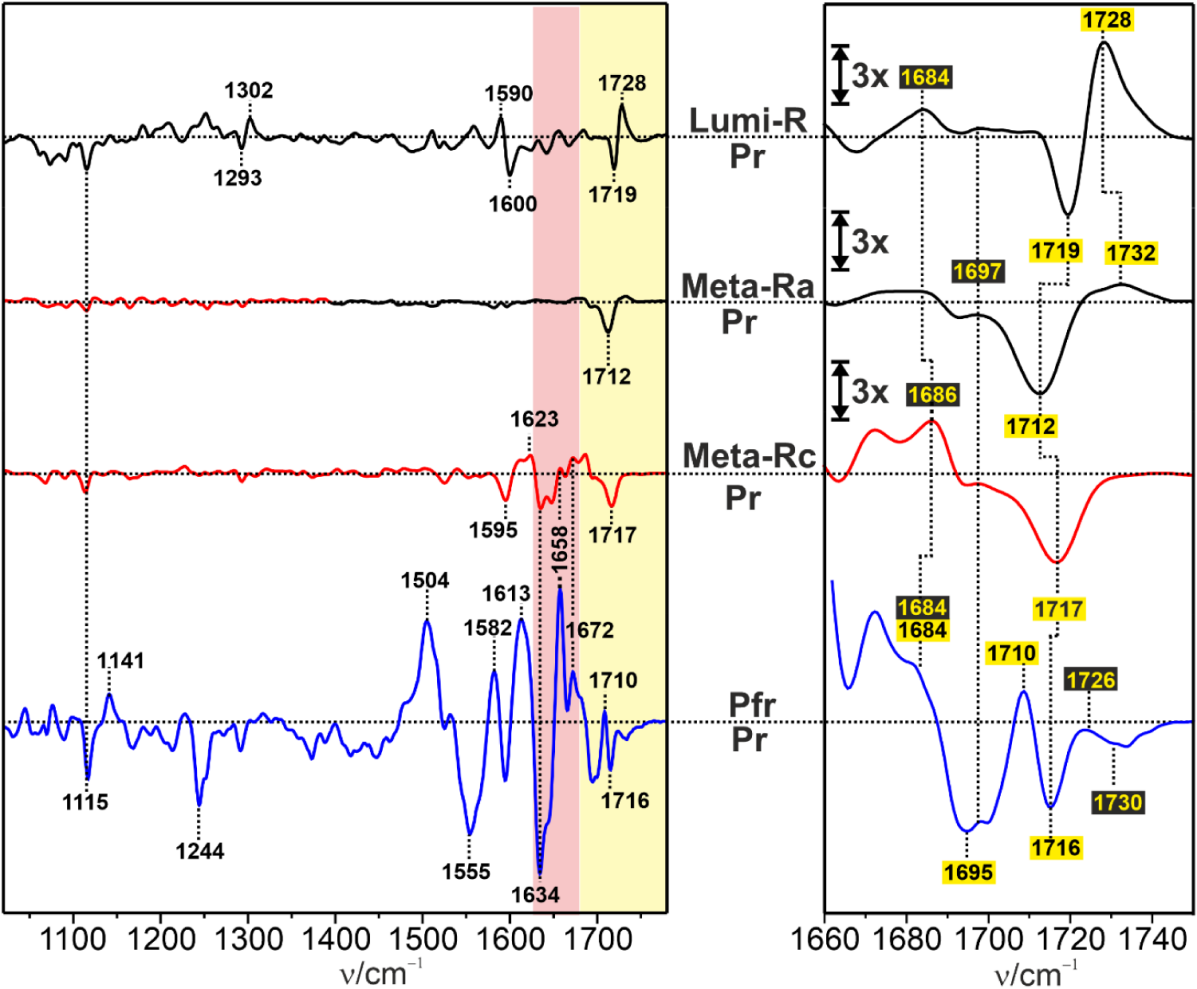
IR difference spectra of the intermediates Lumi-R at 130 K, Meta-Ra at 210 K, Meta-Rc at 250 K, and Pfr at 290 K. In each case, the reference state (negative signals) is Pr. The spectra were scaled to the ring D C=O stretching signal of Pr. The spectra were obtained from Agp1 in H_2_O at pH 7.8. The red and yellow shaded areas indicate the region of the amide I modes of the protein backbone and the carbonyl modes of rings A and D of BV, respectively. The right panel displays a close-up view of the C=O stretching region, with the yellow numbers in black boxes and the black numbers in yellow boxes indicating ring A and D modes, respectively. In this panel, the intensity scale of the “Lumi-R” minus “Pr”, “Meta-Ra” minus “Pr”, and “Meta-Rc” minus “Pr” spectra is enlarged by a factor of 3.

The spectra were measured at the same temperatures used for trapping individual intermediates in the RR experiments. However, unlike the RR spectroscopy, subtraction of contributions from the preceding or subsequent intermediates is not possible in the IR experiments, such that the respective IR difference spectra reflect largely, albeit not exclusively, the indicated intermediates.

In Lumi-R, the spectral changes are restricted to the isomerization site and the ring D C=O group.^56^ This is also true for the subsequent intermediate Meta-Ra. In the “Meta-Rc” minus “Pr” difference spectrum, we also note small positive (1623 and 1658 cm^–1^) and negative signals (1634 cm^–1^) in the amide I band region, pointing to the onset of the secondary structure transition of the tongue from β-sheet/hairpin (1634 cm^–1^) to α-helix/coil (1623 and 1658 cm^–1^).^11,39,59^ This conformational change is completed in the final transition to Pfr, as documented by the strong increase in the signal intensity in this region.

In contrast to the amide I band changes, the ring A and D carbonyl groups undergo structural and environmental changes starting already in Lumi-R. Whereas the C=O modes of Pr, Lumi-R, and Pfr have been thoroughly analyzed in a previous study,^47,55,56,58,60^ the assignment of these modes in the Meta states is more tentative. In Lumi-R, the ring D C=O mode is observed at 1728 cm^–1^ and shifts slightly to 1732 cm^–1^ in Meta-Ra. For the ring A C=O modes, two bands were identified at ca. 1684 and 1697 cm^–1^ corresponding to two substates. Both bands remain largely unchanged from Pr up to the Meta-Rc state. Only in Pfr, the 1697 cm^–1^ band has nearly disappeared in favor of a band at 1726 cm^–1^. In Meta-Rc, however, a positive signal of the ring D C=O stretching is not observed anymore, in contrast to the corresponding negative signal of Pr. We checked this assignment by comparison with the spectra of Agp1 assembled with a BV chromophore that was deuterated at the A–B and C–D methine bridges and at the terminal positions of the ring D vinyl group (Figure 4). According to RR data for Pr and normal-mode analyses within the framework of quantum mechanics/molecular mechanics (QM/MM) calculations,^58^ the ring A C=O frequency does not respond to this isotopic labeling, whereas the ring D C=O stretching displays a small frequency downshift, which is, in fact, in line with assignments discussed above.

**Figure 4:**
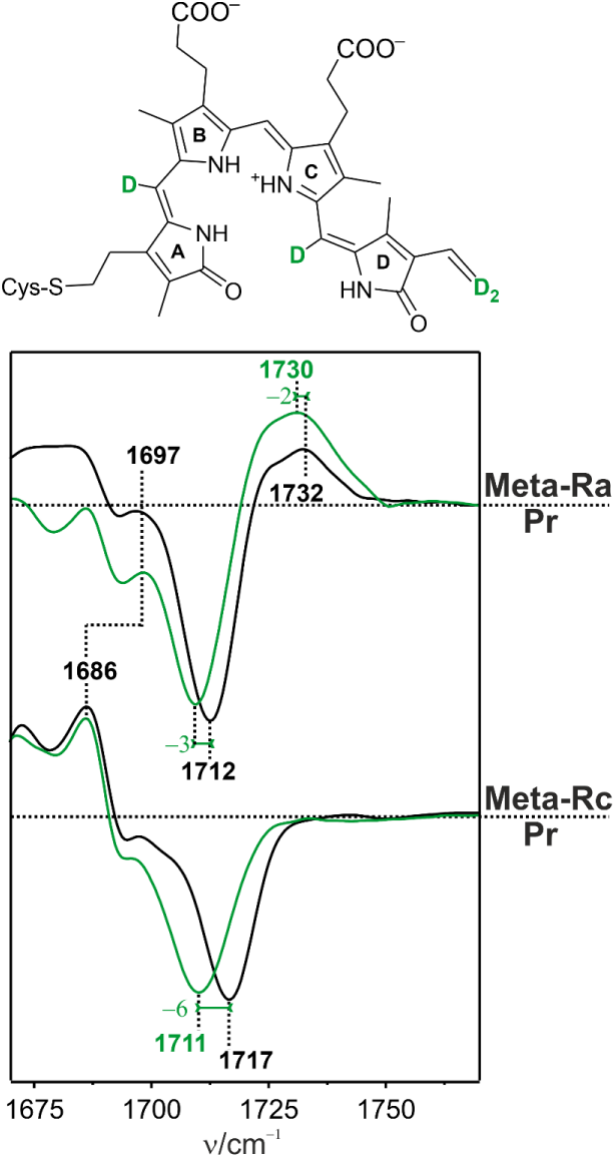
IR difference spectra of “Meta-Ra” minus “Pr” and “Meta-Rc” minus “Pr” in the C=O stretching region. The black and green traces refer to Agp1 assembled with the nonlabeled and deuterium-labeled BV, respectively. The labeling pattern is shown in the structural formula on top.

In Pfr, the C=O stretching of ring D is observed again, albeit shifted to 1710 cm^–1^ (Figure 3). As for Pr, also in Pfr, we note two bands due to each of these modes, implying that in both parent states, two or more substates exist that differ with respect to the conformation or hydrogen bonding interactions of these substituents. This finding is not surprising, as similar structural heterogeneity, particularly of the parent states, has been observed for plant, cyanobacterial, and other bacterial phytochromes.^41,53,54,61,62^

### Keto–Enol Tautomerism in the Meta States

The C=O stretching of ring D is one of the most intense IR-active modes of the tetrapyrrole chromophore. Its disappearance in Meta-Rc implies the loss of the carbonyl function at this ring, which can only be rationalized in terms of an enolization.^39^ Consequently, the keto form shown in Figure 5A (highlighted in yellow), which adequately describes the spectroscopic results of all other intermediate states and Pfr, does not hold for Meta-Rc. Instead, the enol tautomers in Figure 5C and D are both consistent with the IR spectrum, whereas the loss of the N–H ip bending of rings B and C in the RR spectrum of Meta-Rc (*vide supra*) is compatible with the structures in Figure 5B (ring B- or ring C-deprotonated in B_2_ and B_1_, respectively) as suggested previously,^48^ as well as Figure 5D. Hence, only the structure in Figure 5D satisfies both the RR and IR observations.

**Figure 5:**
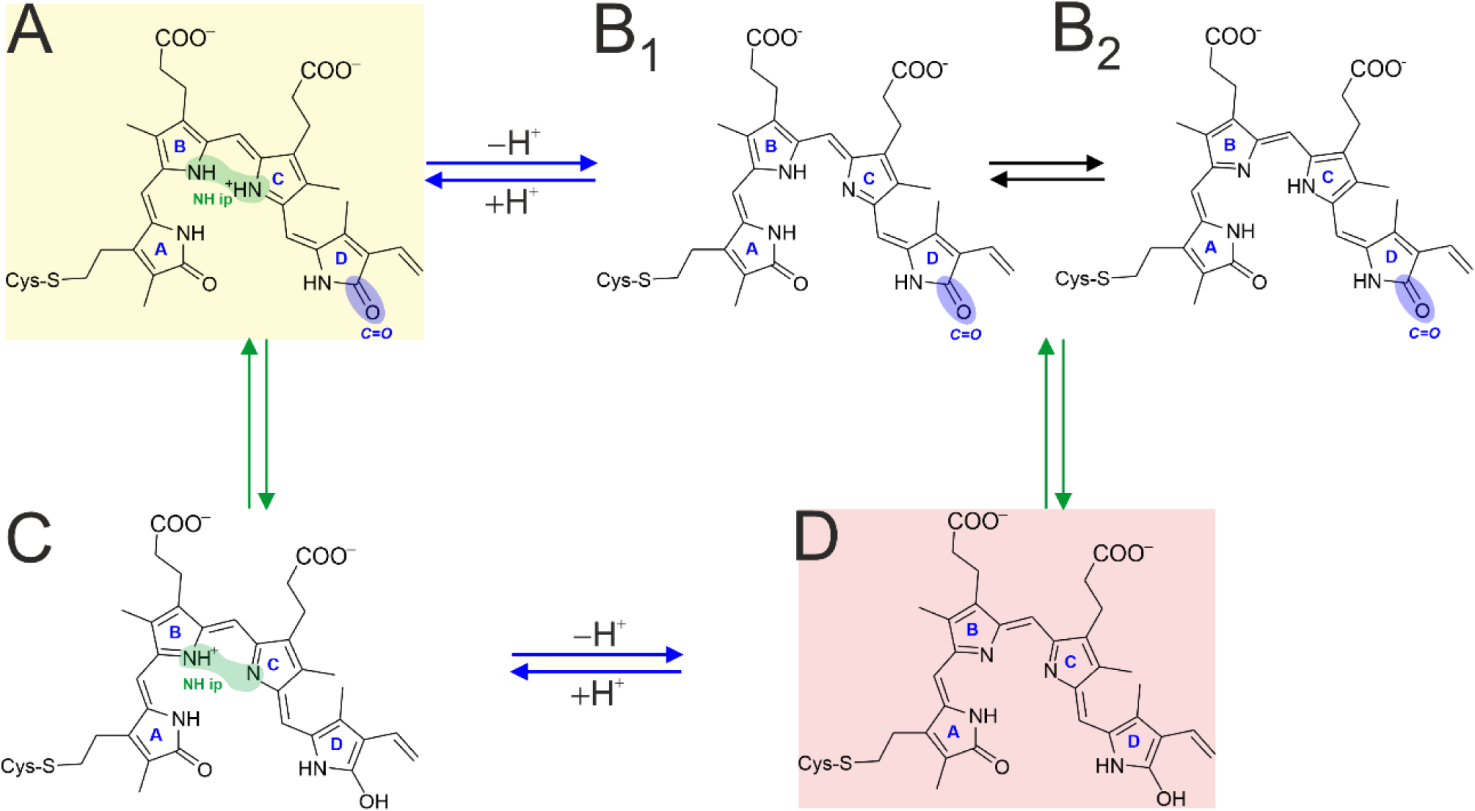
Structural formulas of the tautomerism of the BV chromophore in the Meta-R states of Agp1. The keto tautomer **A**, assigned to Meta-Ra, and the enol tautomer **D,** proposed for Meta-Rc, are highlighted in yellow and red, respectively. The keto structure in **B** may exist in two tautomers that are deprotonated at either ring C (**B_1_**) or ring B (**B_2_**). Note that the enol tautomers in **C** and **D** may be represented by two mesomeric forms with interchanged double/single bond character of the C–D methine bridge. Here only one mesomeric structure is shown. The existence of characteristic RR (NH ip in green) and IR bands (C=O in blue) is indicated in the individual structures.

### Structural Changes as a Function of the pH

We subsequently analyzed the effect of pH changes on the structure of Meta-Rc and Pfr. In Pfr and Pr, lowering the pH from 7.8 to 5.9 does not affect the IR difference spectra in terms of frequencies or band intensities (Figure 6).

**Figure 6:**
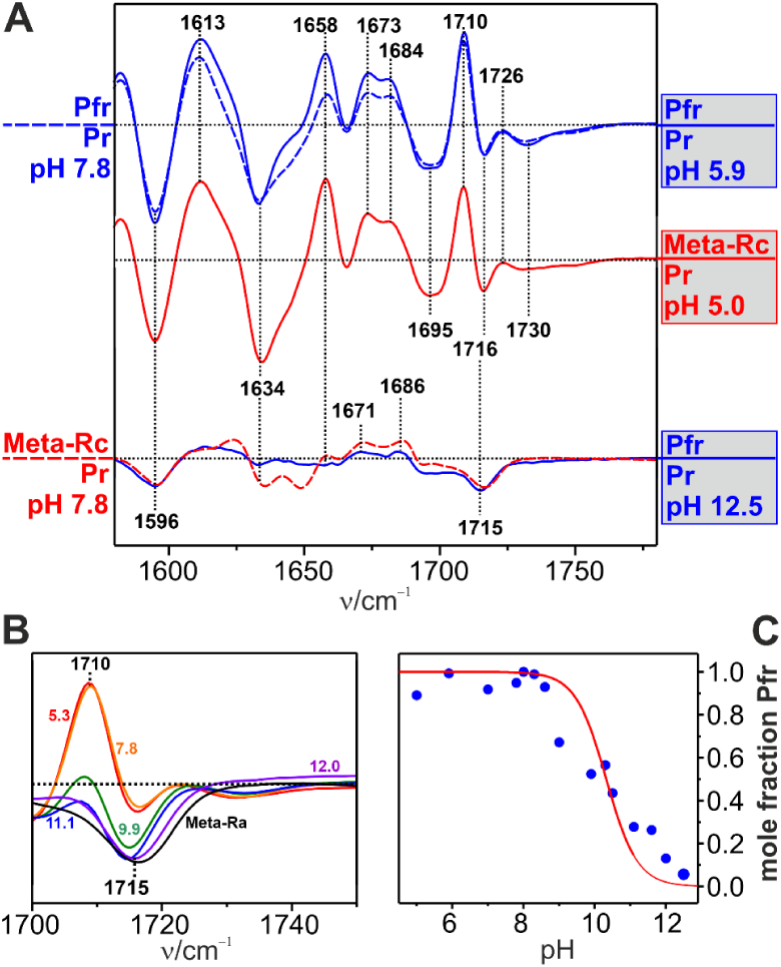
**A**, IR difference spectra “Meta-Rc” minus “Pr” and “Pfr” minus “Pr” at different pH values between 12.5 and 5.0. The solid blue and red lines refer to Pfr and Meta-Rc as the initial states to which the pH changes were applied to. The resultant species are labeled in gray boxes to distinguish them from the reference states at pH 7.8, the spectra of which are represented by dashed traces. **B**, Close-up view of the ring D C=O stretching region of “Pfr” minus “Pr” difference spectra as a function of the pH, indicated by numbers in the same color as the corresponding lines. **C**, Quantitative evaluation of the pH dependence using the ring D C=O stretching difference intensity in **B** as a measure for the Pfr mole fraction. The data (blue closed circles) was normalized to the maximum value (pH 6, mole fraction 1.0). The red line represents a fit of the Henderson–Hasselbalch equation to the data, yielding a p*K*_A_ of 10.3.

In contrast, lowering the pH to 5.0 in the Meta-Rc state (250 K) leads to strong difference signals in the amide I region, very similar to that of Pfr at pH 7.8. Moreover, also the C=O stretching of ring D at 1710 cm^–1^ is fully recovered. On the other hand, upon increasing the pH to 12.5 in the Pfr state (290 K), the amide I signals as well as the positive band of the ring D C=O stretching completely disappear, and the spectrum becomes very similar to that of Meta-Rc.^47^ These results show that with respect to the protein structure and the interactions with the BV carbonyl groups, Meta-Rc and Pfr can be converted into each other by varying the pH, regardless of the temperature in the range between 250 and 290 K. Using the ring D C=O stretching signal as a measure for the Pfr mole fraction, we fitted the Henderson–Hasselbalch equation to the data and obtained a p*K*_A_ value of 10.3 for the transition at 290 K. Note that the deviations from the fitted function are due to the uncertainty in the signal determination, particularly at weak signals.

In a similar way, we studied the pH dependence by RR spectroscopy, which offers a similar, albeit not identical, picture to the IR experiments (Figure 7). In the case of Pfr, the spectra remain largely unchanged in acidic solution (pH 7.8 vs 5.8), except for minor frequency upshifts of the characteristic marker bands by 2–3 cm^–1^. In contrast, upon lowering the pH to 6.0 in the Meta-Rc state, bands appear that can readily be related to the HOOP, N–H ip, and C–D stretching modes of Pfr. However, this spectrum does not reflect a mixture of Pfr and Meta-Rc as captured at pH 7.8, since attempts to subtract contributions of one of these states immediately cause artifacts. Thus, this spectrum reflects a new state that is different from Meta-Rc and Pfr.

**Figure 7:**
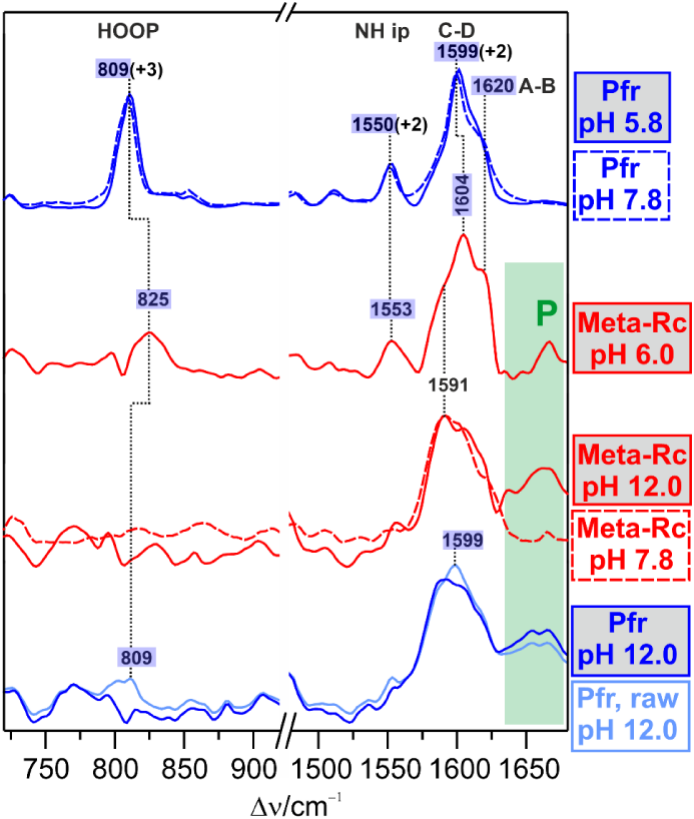
RR spectra of Pfr (blue) and Meta-Rc (red) at different pH. The species generated by pH above and below 7.8 are labeled in gray boxes to distinguish them from the reference states at pH 7.8, the spectra of which are represented by dashed traces. The light blue trace at the bottom is the raw spectrum of Pfr at pH 12, and the dark blue line is the pure spectrum of this state after subtracting a residual contribution of the reference Pfr at pH 7.8. Characteristic bands of Pfr are marked with blue rectangles. The green rectangle indicates nonresonant Raman bands of the protein (P).

Shifting the pH toward an alkaline solution has an opposite effect on the spectra of the two states. Whereas the Meta-Rc spectrum remains unchanged upon raising the pH to 12, in Pfr we note a distinct decrease in the characteristic bands of Pfr, and the spectrum resembles that of Meta-Rc at pH 7.8. After subtracting the residual contribution of the reference state of Pfr (pH 7.8) from the raw spectrum of Pfr, the resultant spectrum (Pfr at pH 12.0) is essentially the same as that of Meta-Rc at pH 7.8.

## Discussion

### Keto–Enol Tautomerism in Meta-Rc

The transition from Meta-Ra to Meta-Rc is associated with the transition from the classical keto form to the enol tautomer (Figure 5). Tautomerism is attributed to ring D on the basis of the assignment of the C=O stretching modes in the IR difference spectra. In addition, the enol tautomer formed upon proton transfer to the ring D carbonyl allows for a mesomeric electron distribution in which the single- and double-bond character at the C–D methine bridge is interchanged. This facilitates the rotation of ring D as a prerequisite for the thermal back reaction directly to the initial Pr state. This shortcut reaction pathway in Meta-Rc has been identified previously.^44^ Enolization of the ring D carbonyl has been postulated already decades ago,^2,63^ but only recently has the first experimental evidence for this mechanism been documented in the case of the thermal back reaction from Pr to Pfr in bathy phytochromes.^39^ The present findings for Meta-Rc of Agp1 represent a second example, such that one may consider enolization and subsequent single-bond rotation of the methine bridge as a general mechanism of thermal isomerization in phytochromes, although the reacting enolic state may not be identified in each case. Note that a high steady-state concentration of the enol tautomer, required for a spectroscopic detection, is not essential for an efficient reaction, which in turn primarily depends on the reactivity of this tautomer.

### Chromophore Enolization and Proton Transfer in Meta-Rc

The formation of Meta-Rc has been shown to be coupled with the proton transfer from the protein to the solution phase, where it can be detected by a pH-sensitive indicator dye.^48^ Proton reuptake takes place with the formation of Pfr. This reversible proton transfer is most likely a general property of the photoconversion from Pr to Pfr since it was observed not only for Agp1 but also for the cyanobacterial phytochrome Cph1.^53,64,65^ Among the involved amino acid residues in the chromophore binding pocket, the conserved His250 is important for proton reuptake but not critical for proton release, which is also observed upon substituting His250 by Gln.^48,49^

According to NMR analyses, the Pr state of Cph1 forms an equilibrium of two substates (Pr–I, Pr–II) that differ by the protonation state of His260, the counterpart of His250 in Agp1.^32,66,67^ The transition between the two substates thus corresponds to the (de)­protonation of His260, which is double-protonated (cationic) in Pr–I but single-protonated in Pr–II. The p*K*_A_ of this transition was determined to be around 7.7 ± 0.2.^53^ The second highly conserved His290 (His280 in Agp1) is single-protonated in both states. In view of the similar structural heterogeneity of the Pr states in Cph1 and Agp1,^53,58^ the same protonation pattern of the conserved His residues probably exists in Agp1. Specifically, a p*K*_A_ of His250 around physiological pH is consistent with the originally suggested role of this residue in proton translocation.^48^ The transition from Meta-Ra to Meta-Rc is accompanied by the enolization of the chromophore (Figure 5; A→C), which promotes the transfer of a proton to His250. This interpretation is in line with the assignment of the Meta-Rc chromophore to a deprotonated enolic BV (Figure 5, structure D) as derived from the RR and IR data. Thus, the cationic His250 may act as the first link in the proton translocation chain from BV to the solution phase by transferring a proton to the subsequent proton acceptor and accepting the proton from BV in a Grotthuss-type transport mechanism.^68^ This interpretation is supported by mutational studies on Cph1Δ2 and the bacterial phytochrome *Dr*BphP from *Deinococcus radiodurans*, which underline the importance of His250 for the protonation state of the chromophore.^64,69^ Substitution of this residue by Gln in Cph1 and by Ala in *Dr*BphP substantially lowers the p*K*_A_ of the protonated chromophore from ca. 10 to a value closer to neutral pH. The tight proton-linked coupling of this His residue with the chromophore has also been documented in a recent ultrafast crystallographic study, which specifically showed the proton-dependent assistance of the C–D methine bridge isomerization.^70^

### Proton-Coupled Chromophore and Protein Structural Changes

Concomitant with the enolization of the chromophore in Meta-Rc, we note very small but unambiguous signals in the amide I region of the IR difference spectra, indicating the onset of the tongue restructuring. This situation is reminiscent of the destabilization of the tongue structure in Meta-F of the bathy phytochrome Agp2.^22^ The complete conversion of the tongue from the β-sheet/hairpin to an α-helix/coil structure occurs with the transition from Meta-Rc to Pfr. Thus, we conclude that proton release from the chromophore represents the start signal for tongue restructuring, but its completion requires chromophore reprotonation. This raises the question of how proton transfer, secondary structure transformation, and chromophore structural changes are coupled.

At any constant temperature between 250 and 290 K, the tongue forms a pH-dependent conformational equilibrium between the β-sheet/hairpin and α-helix/coil structure, with the latter prevailing at low pH. At 290 K and pH 7.8, where Pfr prevails and the α-helix/coil tongue is the stable conformation, the p*K*_A_ of this transition is close to 11. Conversely, at 250 K and pH 7.8, where Meta-Rc exists and the β-sheet/hairpin tongue is the stable conformation, the p*K*_A_ is distinctly lower, presumably near 6.

The chromophore conformational changes, which are reflected by the RR spectra, cannot be grouped into the two-state tongue structure scheme as derived from the IR difference spectra. Starting with Pfr, the shift to pH 12 leads to a chromophore structure that is essentially the same as that in Meta-Rc at pH 7.8, indicating a deprotonated enol chromophore. However, acidification of Meta-Rc from pH 7.8 to 6.0 leads to a protonated keto tautomer with a perturbed tetrapyrrole geometry that differs from that of Pfr at pH 7.8. The state, which we denote as pre-Pfr, is characterized by upshifts of the HOOP mode from 809 to 825 cm^–1^ and the C–D stretching mode from 1599 to 1604 cm^–1^. These spectral changes indicate a reduced dihedral angle at the C–D methine bridge single bond and an increased dihedral angle at the corresponding double bond, leading to modified twist angle of the C–D methine bridge.^54^ Most likely, this state adopts a distorted *ZZEssa* chromophore geometry, which relaxes in the last step of the reaction sequence to Pfr.

Figure 8 shows a reaction scheme for the pathway from Meta-Ra to Pfr. The scheme takes into account back reactions according to the principle of microscopic reversibility. At pH 7.8, the back reactions can largely be neglected, but they gain importance upon increasing the pH such that the equilibria can be shifted back to Meta-Rc even at 290 K. At pH 7.8, the reaction sequence is blocked at Meta-Ra and Meta-Rc at 210 and 250 K, respectively. The pre-Pfr state cannot be trapped at pH 7.8, most likely since its decay is faster than its formation. The situation is different when the pH is lowered. Then the reaction cascade is blocked at pre-Pfr at 250 K. The scheme in Figure 8 implies that Pfr can be thermally converted to the Meta-R states upon restructuring of the tongue, corresponding to the inactivation of the photoreceptor. This reaction may be the basis for the previously observed activity of lowering Pfr in Agp1 upon increasing the temperature.^71^ Interestingly, this effect has also been noted for other phytochromes, pointing to their capability to act as a light- and temperature sensor.^72^

**Figure 8:**
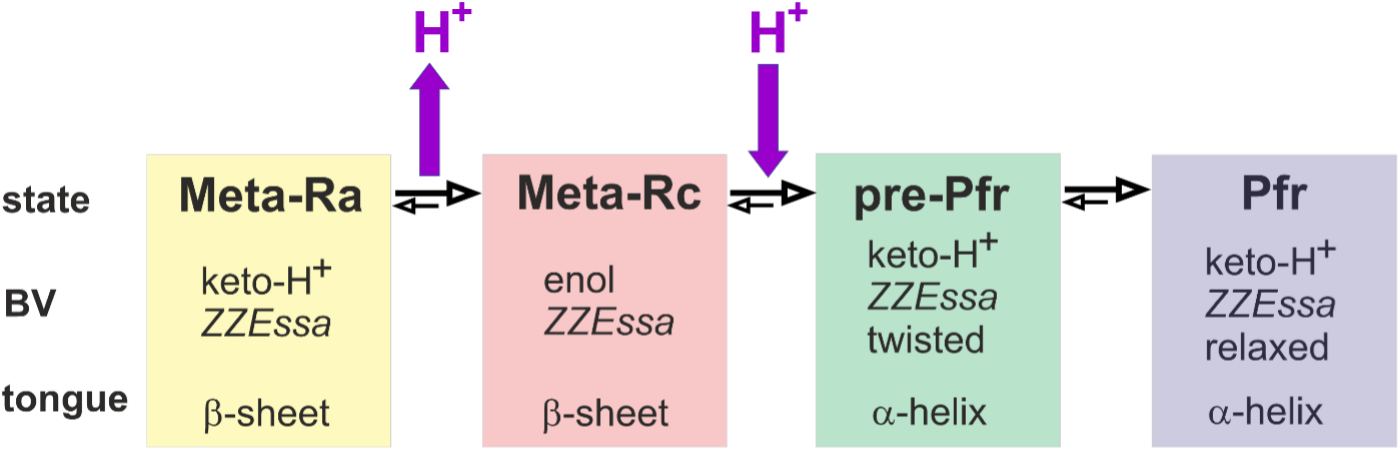
Schematic representation of the reaction sequence from Meta-Rc to Pfr, including a description of the structural changes associated with the individual reaction steps.

In addition to the kinetic description, the scheme in Figure 8 also reflects the coupling of proton transfer with protein and chromophore structural changes. The transition from Meta-Ra to Meta-Rc is associated with the enolization and deprotonation of the BV. This reorganization in the chromophore binding pocket is sensed by the tongue, which is partially destabilized but stays in the β-sheet structure. The subsequent reuptake of a proton leads to the reprotonation of BV. Thus, the keto tautomer is restored, and the chromophore adopts a *ZZEssa* conformation with a twisted C–D methine bridge double bond. Accordingly, we suggest that the structural and electrostatic changes trigger the α-helix formation of the tongue. In this pre-Pfr state, the tongue has adopted its equilibrium structure, whereas the chromophore requires an additional conformational adjustment to reach a more relaxed C–D methine bridge geometry in the final reaction step to Pfr.

## Conclusions

The spectroscopic analysis of Meta-Rc provides new insight into its function as a central hub for transmitting the signal of chromophore photoisomerization to photoreceptor activation. First, due to enolization of the ring D carbonyl that facilitates thermal *Z/E* isomerization, Meta-Rc opens an alternative but nonproductive reaction route back to the initial dark state Pr.^44^ Second, in the productive pathway, proton translocation in Meta-Rc is a critical trigger for protein structural changes. Although this could be demonstrated only for the last reaction step (pre-Pfr→Pfr), it is plausible that, throughout the photoconversion, proton transfer and structural rearrangements at the chromophore and the tongue occur in an alternating, stepwise manner. This mechanism probably also holds for the Pr→Pfr conversion of other prototypical and bathy phytochromes since they all run through a Meta-Rc intermediate with similar spectral properties.^10,11,55,73^ The reverse Pfr→Pr transformation is also coupled to proton translocation, albeit via a different mechanism, as demonstrated for bathy phytochromes.^11,39,74^ Thus, it is very likely that proton transfer and the functional secondary structure transition of the tongue do not just accidentally take place in parallel but are linked in a cause-effect relationship. This relationship may be based on the substantial change in the local electric field that is associated with the movement of a positive charge and may stabilize or destabilize the β-sheet or α-helical structure of the tongue.^75–77^

## Data Availability

The data that support the findings of this study are available from the corresponding author.

## References

[1] Hughes J., Winkler A. (2024). New Insight Into Phytochromes: Connecting Structure
to Function. Annu. Rev. Plant Biol..

[2] Rockwell N. C., Su Y.-S., Lagarias J. C. (2006). Phytochrome
Structure and Signaling
Mechanisms. Annu. Rev. Plant Biol..

[3] Burgie E. S., Vierstra R. D. (2014). Phytochromes: An
atomic perspective on photoactivation
and signaling. Plant Cell.

[4] Gourinchas G., Etzl S., Winkler A. (2019). Bacteriophytochromes – from
informative model systems of phytochrome function to powerful tools
in cell biology. Curr. Opin. Struct. Biol..

[5] Hughes J., Lamparter T., Mittmann F., Hartmann E., Gärtner W., Wilde A., Börner T. (1997). A prokaryotic phytochrome. Nature.

[6] Corrochano L. M., Gutiérrez G., Corrochano-Luque M., Franco-Cano A., Cánovas D. (2025). How fungi
see the world: Fungal photoreceptors and
their role in the regulation of fungal biology. Microbiol. Mol. Biol. Rev..

[7] Lamparter T., Krauß N., Scheerer P. (2017). Phytochromes from Agrobacterium fabrum. Photochem. Photobiol..

[8] Rockwell N. C., Lagarias J. C. (2020). Phytochrome evolution in 3D: Deletion, duplication,
and diversification. New Phytol..

[9] Sineshchekov V. A. (1995). Photobiophysics
and photobiochemistry of the heterogeneous phytochrome system. Biochim. Biophys. Acta.

[10] Hildebrandt P. (2023). Vibrational
Spectroscopy of Phytochromes. Biomolecules.

[11] Fernández
López M., Dahl M., Velázquez Escobar F., Bonomi H. R., Kraskov A., Michael N., Mroginski M. A., Scheerer P., Hildebrandt P. (2022). Photoinduced reaction mechanisms
in prototypical and bathy phytochromes. Phys.
Chem. Chem. Phys..

[12] Takala H., Björling A., Berntsson O., Lehtivuori H., Niebling S., Hoernke M., Kosheleva I., Henning R., Menzel A., Ihalainen J. A., Westenhoff S. (2014). Signal amplification and transduction in phytochrome
photosensors. Nature.

[13] Gourinchas G., Etzl S., Göbl C., Vide U., Madl T., Winkler A. (2017). Long-range allosteric
signaling in red light–regulated
diguanylyl cyclases. Sci. Adv..

[14] Gourinchas G., Heintz U., Winkler A. (2018). Asymmetric activation mechanism of
a homodimeric red light-regulated photoreceptor. Elife.

[15] Björling A., Berntsson O., Lehtivuori H., Takala H., Hughes A. J., Panman M., Hoernke M., Niebling S., Henry L., Henning R. (2016). Structural
photoactivation of a full-length bacterial
phytochrome. Sci. Adv..

[16] Auldridge M. E., Forest K. T. (2011). Bacterial phytochromes: More than meets the light. Crit. Rev. Biochem. Mol. Biol..

[17] Karniol B., Vierstra R. D. (2003). The pair of bacteriophytochromes
from Agrobacterium
tumefaciens are histidine kinases with opposing photobiological properties. Proc. Natl. Acad. Sci. U.S.A..

[18] Wagner J. R., Brunzelle J. S., Forest K. T., Vierstra R. D. (2005). A light-sensing
knot revealed by the structure of the chromophore-binding domain of
phytochrome. Nature.

[19] Essen L.-O., Mailliet J., Hughes J. (2008). The structure
of a complete phytochrome
sensory module in the Pr ground state. Proc.
Natl. Acad. Sci. U. S. A..

[20] Yang X., Kuk J., Moffat K. (2008). Crystal structure of Pseudomonas aeruginosa bacteriophytochrome:
Photoconversion and signal transduction. Proc.
Natl. Acad. Sci. U. S. A..

[21] Yang X., Ren Z., Kuk J., Moffat K. (2011). Temperature-scan cryocrystallography
reveals reaction intermediates in bacteriophytochrome. Nature.

[22] Schmidt A., Sauthof L., Szczepek M., Lopez M. F., Velazquez
Escobar F., Qureshi B. M., Michael N., Buhrke D., Stevens T., Kwiatkowski D. (2018). Structural snapshot
of a bacterial phytochrome in its functional intermediate state. Nat. Commun..

[23] Burgie E. S., Zhang J., Vierstra R. D. (2016). Crystal
Structure of Deinococcus
Phytochrome in the Photoactivated State Reveals a Cascade of Structural
Rearrangements during Photoconversion. Structure.

[24] Sauthof L., Szczepek M., Schmidt A., Bhowmick A., Dasgupta M., Mackintosh M. J., Gul S., Fuller F. D., Chatterjee R., Young I. D. (2025). Serial-femtosecond
crystallography reveals
how a phytochrome variant couples chromophore and protein structural
changes. Sci. Adv..

[25] Carrillo M., Pandey S., Sanchez J., Noda M., Poudyal I., Aldama L., Malla T. N., Claesson E., Wahlgren W. Y., Feliz D. (2021). High-resolution crystal
structures of transient intermediates
in the phytochrome photocycle. Structure.

[26] Malla T. N., Aldama L., Leon V., Feliz D., Hu H., Thomas I., Cellini A., Wahlgren W. Y., Nimmrich A., Botha S. (2025). Observation
of early events in the photoactivation
of Myxobacterial phytochrome using time-resolved serial femtosecond
crystallography. Commun. Chem..

[27] Claesson E., Wahlgren W. Y., Takala H., Pandey S., Castillon L., Kuznetsova V., Henry L., Panman M., Carrillo M., Kübel J. (2020). The primary structural
photoresponse of phytochrome
proteins captured by a femtosecond x-ray laser. Elife.

[28] Sanchez J. C., Carrillo M., Pandey S., Noda M., Aldama L., Feliz D., Claesson E., Wahlgren W. Y., Tracy G., Duong P. (2019). High-resolution crystal
structures of a myxobacterial
phytochrome at cryo and room temperatures. Struct.
Dyn..

[29] Edlung P., Takala H., Claesson E., Henry L., Dods R., Lehtivuori H., Panman M., Pande K., White T., Nakane T. (2016). The room temperature crystal structure of a bacterial
phytochrome determined by serial femtosecond crystallography. Sci. Rep..

[30] Rohmer T., Lang C., Bongards C., Gupta K. B. S. S., Neugebauer J., Hughes J., Gärtner W., Matysik J. (2010). Phytochrome as molecular machine: Revealing chromophore
action during the Pfr → Pr photoconversion by magic-angle spinning
NMR spectroscopy. J. Am. Chem. Soc..

[31] Stöppler D., Song C., van Rossum B. J., Geiger M. A., Lang C., Mroginski M. A., Jagtap A. P., Sigurdsson S. T., Matysik J., Hughes J., Oschkinat H. (2016). Dynamic Nuclear
Polarization Provides New Insights into Chromophore Structure in Phytochrome
Photoreceptors. Angew. Chemie - Int. Ed..

[32] Song C., Mroginski M. A., Lang C., Kopycki J., Gärtner W., Matysik J., Hughes J. (2018). 3D structures of plant phytochrome
a as Pr and Pfr from solid-state NMR: Implications for molecular function. Front. Plant Sci..

[33] Singer P., Wörner S., Lamparter T., Diller R. (2016). Spectroscopic Investigation
on the Primary Photoreaction of Bathy Phytochrome Agp2-Pr of Agrobacterium
fabrum: Isomerization in a pH-dependent H-bond Network. ChemPhyschem.

[34] Gottlieb S. M., Kim P. W., Rockwell N. C., Hirose Y., Ikeuchi M., Lagarias J. C., Larsen D. S. (2013). Primary photodynamics of the green/red-absorbing
photoswitching regulator of the chromatic adaptation e domain from
fremyella diplosiphon. Biochemistry.

[35] Yang Y., Linke M., Von Haimberger T., Hahn J., Matute R., González L., Schmieder P., Heyne K. (2012). Real-time tracking
of phytochrome’s orientational changes during Pr photoisomerization. J. Am. Chem. Soc..

[36] Yang Y., Stensitzki T., Sauthof L., Schmidt A., Piwowarski P., Velazquez Escobar F., Michael N., Nguyen A. D., Szczepek M., Brünig F. N., Netz R. R., Mroginski M. A., Adam S., Bartl F., Schapiro I., Hildebrandt P., Scheerer P., Heyne K. (2022). Ultrafast proton-coupled isomerization
in the phototransformation of phytochrome. Nat.
Chem..

[37] Ihalainen J. A., Gustavsson E., Schroeder L., Donnini S., Lehtivuori H., Isaksson L., Thöing C., Modi V., Berntsson O., Stucki-Buchli B., Liukkonen A., Häkkänen H., Kalenius E., Westenhoff S., Kottke T. (2018). Chromophore-Protein
Interplay during the Phytochrome Photocycle Revealed by Step-Scan
FTIR Spectroscopy. J. Am. Chem. Soc..

[38] Lenngren N., Edlund P., Häkkänen H., Takala H., Stucki-Buchli B., Rumfeldt J., Peshev I., Westenhoff S., Ihalainen J. A. (2018). Coordination of the biliverdin D-ring
in bacteriophytochromes. Phys. Chem. Chem. Phys..

[39] Velazquez
Escobar F., Piwowarski P., Salewski J., Michael N., Fernandez Lopez M., Rupp A., Qureshi B. M., Scheerer P., Bartl F., Frankenberg-Dinkel N. (2015). A protonation-coupled
feedback mechanism controls the signalling process in bathy phytochromes. Nat. Chem..

[40] Hildebrandt P. (2023). Vibrational
Spectroscopy of Phytochromes. Biomolecules.

[41] Nagano S., Song C., Rohr V., Mackintosh M. J., Hoang O. T., Kraskov A., Yang Y., Hughes J., Heyne K., Mroginski M. A., Schapiro I., Hildebrandt P. (2025). Integrated
Study of Fluorescence Enhancement in the Y176H Variant of Cyanobacterial
Phytochrome Cph1. Biochemistry.

[42] Takala H., Lehtivuori H. K., Berntsson O., Hughes A., Nanekar R., Niebling S., Panman M., Henry L., Menzel A., Westenhoff S., Ihalainen J. A. (2018). On the (un)­coupling of the chromophore,
tongue interactions, and overall conformation in a bacterial phytochrome. J. Biol. Chem..

[43] Fischer A. J., Rockwell N. C., Jang A. Y., Ernst L. A., Waggoner A. S., Duan Y., Lei H., Lagarias J. C. (2005). Multiple
roles of
a conserved GAF domain tyrosine residue in cyanobacterial and plant
phytochromes. Biochemistry.

[44] Buhrke D., Kuhlmann U., Michael N., Hildebrandt P. (2018). The Photoconversion
of Phytochrome Includes an Unproductive Shunt Reaction Pathway. ChemPhyschem.

[45] Merga G., Fernandez Lopez M., Fischer P., Piwowarski P., Nogacz Z., Kraskov A., Buhrke D., Velazquez
Escobar F., Michael N., Síebert F. (2021). Light- and Temperature-dependent Dynamics of Chromophore and Protein
Structural Changes in Bathy Phytochrome Agp2. Phys. Chem. Chem. Phys..

[46] Schwinté P., Gärtner W., Sharda S., Mroginski M.-A., Hildebrandt P., Siebert F. (2009). The photoreactions of recombinant
phytochrome CphA from the cyanobacterium Calothrix PCC7601: A low-temperature
UV-Vis and FTIR study. Photochem. Photobiol..

[47] Piwowarski P., Ritter E., Hofmann K.-P., Hildebrandt P., von Stetten D., Scheerer P., Michael N., Lamparter T., Bartl F. (2010). Light-Induced Activation of Bacterial Phytochrome Agp1Monitored by
Static and Time-Resolved FTIR Spectroscopy. ChemPhyschem.

[48] Borucki B., von Stetten D., Seibeck S., Lamparter T., Michael N., Mroginski M. A., Otto H., Murgida D. H., Heyn M. P., Hildebrandt P. (2005). Light-induced Proton Release of Phytochrome
Is Coupled to the Transient Deprotonation of the Tetrapyrrole Chromophore. J. Biol. Chem..

[49] Von
Stetten D., Seibeck S., Michael N., Scheerer P., Mroginski M. A., Murgida D. H., Krauss N., Heyn M. P., Hildebrandt P., Borucki B., Lamparter T. (2007). Highly conserved
residues Asp-197 and His-250 in Agp1 phytochrome control the proton
affinity of the chromophore and Pfr formation. J. Biol. Chem..

[50] Lamparter T., Michael N. (2005). Agrobacterium Phytochrome as an Enzyme for the Production
of ZZE Bilins. Biochemistry.

[51] Kŗezel A., Bal W. (2004). A formula for correlating
pKa values determined in D 2O and H2O. J. Inorg.
Biochem..

[52] Velazquez
Escobar F., Kneip C., Michael N., Hildebrandt T., Tavraz N., Gärtner W., Hughes J., Friedrich T., Scheerer P., Mroginski M. A., Hildebrandt P. (2020). The Lumi-R
Intermediates of Prototypical Phytochromes. J. Phys. Chem. B.

[53] Velazquez
Escobar F., Lang C., Takiden A., Schneider C., Balke J., Hughes J., Alexiev U., Hildebrandt P., Mroginski M. A. (2017). Protonation-dependent structural heterogeneity in the
chromophore binding site of cyanobacterial phytochrome cph1. J. Phys. Chem. B.

[54] Salewski J., Escobar F. V., Kaminski S., Von Stetten D., Keidel A., Rippers Y., Michael N., Scheerer P., Piwowarski P., Bartl F. (2013). Structure of the biliverdin
cofactor in the Pfr state of bathy and prototypical phytochromes. J. Biol. Chem..

[55] Kraskov A., Buhrke D., Scheerer P., Shaef I., Sanchez J., Carrillo M., Noda M., Feliz D., Stojkovic E., Hildebrandt P. (2021). On the Role of the Conserved Histidine at the Chromophore
Isomerization Site in Phytochromes. J. Phys.
Chem. B.

[56] Merga G., Große M., Piwowarski P., Kraskov A., Escobar F. V., Michael N., Ebrahim M., Sauthof L., Scheerer P., Bartl F. (2025). Photoisomerization of phytochromés chromophore:
A vibrational spectroscopic view on the primary ground state processes. RSC Adv..

[57] Kneip C., Hildebrandt P., Schlamann W., Braslavsky S. E., Mark F., Schaffner K. (1999). Protonation
state and structural
changes of the tetrapyrrole chromophore during the Pr → Pfr
phototransformation of phytochrome: A resonance Raman spectroscopic
study. Biochemistry.

[58] Takiden A., Velazquez-Escobar F., Dragelj J., Woelke A. L., Knapp E.-W., Piwowarski P., Bartl F., Hildebrandt P., Mroginski M. A. (2017). Structural
and Vibrational Characterization of the
Chromophore Binding Site of Bacterial Phytochrome Agp1. Photochem. Photobiol..

[59] Stojković E. A., Toh K. C., Alexandre M. T. A., Baclayon M., Moffat K., Kennis J. T. M. (2014). FTIR spectroscopy
revealing light-dependent refolding
of the conserved tongue region of bacteriophytochrome. J. Phys. Chem. Lett..

[60] Van
Thor J. J., Fisher N., Rich P. R. (2005). Assignments of the
Pfr - Pr FTIR difference spectrum of cyanobacterial phytochrome Cph1
using15N and13C isotopically labeled phycocyanobilin chromophore. J. Phys. Chem. B.

[61] Mroginski M. A., Kaminski S., Von Stetten D., Ringsdorf S., Gärtner W., Essen L. O., Hildebrandt P. (2011). Structure
of the chromophore binding pocket in the Pr state of plant phytochrome
phyA. J. Phys. Chem. B.

[62] Velazquez
Escobar F., von Stetten D., Günther-Lütkens M., Keidel A., Michael N., Lamparter T., Essen L.-O., Hughes J., Gärtner W., Yang Y. (2015). Conformational heterogeneity of the Pfr chromophore
in plant and cyanobacterial phytochromes. Front.
Mol. Biosci..

[63] Lagarias J. C., Rapoport H. (1980). Chromopeptides from Phytochrome.
The Structure and
Linkage of the PR Form of the Phytochrome Chromophore. J. Am. Chem. Soc..

[64] Hahn J., Strauss H. M., Landgraf F. T., Gimenèz H. F., Lochnit G., Schmieder P., Hughes J. (2006). Probing protein-chromophore
interactions in Cph1 phytochrome by mutagenesis. FEBS J..

[65] Van
Thor J. J., Borucki B., Crielaard W., Otto H., Lamparter T., Hughes J., Hellingwerf K. J., Heyn M. P. (2001). Light-induced proton release and proton uptake reactions
in the cyanobacterial phytochrome Cph1. Biochemistry.

[66] Song C., Psakis G., Lang C., Mailliet J., Gärtner W., Hughes J., Matysik J. (2011). Two ground
state isoforms and a chromophore
D-ring photoflip triggering extensive intramolecular changes in a
canonical phytochrome. Proc. Natl. Acad. Sci.
U. S. A..

[67] Song C., Essen L. O., Gärtner W., Hughes J., Matysik J. (2012). Solid-state
NMR spectroscopic study of chromophore-protein interactions in the
Pr ground state of plant phytochrome A. Mol.
Plant.

[68] Cukierman S. (2006). Et tu, Grotthuss!
and other unfinished stories. Biochim. Biophys.
Acta - Bioenerg..

[69] Rumfeldt J. A., Takala H., Liukkonen A., Ihalainen J. A. (2019). UV-Vis
Spectroscopy Reveals a Correlation Between Y263 and BV Protonation
States in Bacteriophytochromes. Photochem. Photobiol..

[70] Shankar M. K., Grunewald L., Wahlgren W. Y., Stucki-Buchli B., Nimmrich A., Kurttila M., Fischer A. L., Salvadori G., Cellini A., Maj P. (2025). Ultrafast, remote-controlled
protonation reaction enables structural changes in a phytochrome. Sci. Adv..

[71] Njimona I., Lamparter T. (2011). Temperature
effects on agrobacterium phytochrome agp1. PLoS
One.

[72] Njimona I., Yang R., Lamparter T. (2014). Temperature
effects on bacterial
phytochrome. PLoS One.

[73] Wagner J. R., Zhang J., von Stetten D., Günther M., Murgida D. H., Mroginski M. A., Walker J. M., Forest K. T., Hildebrandt P., Vierstra R. D. (2008). Mutational Analysis of Deinococcus
radiodurans Bacteriophytochrome Reveals Key Amino Acids Necessary
for the Photochromicity and Proton Exchange Cycle of Phytochromes. J. Biol. Chem..

[74] Velázquez
Escobar F., Buhrke D., Michael N., Sauthof L., Wilkening S., Tavraz N. N., Salewski J., Frankenberg-Dinkel N., Mroginski M. A., Scheerer P., Friedrich T., Siebert F., Hildebrandt P. (2017). Common Structural Elements in the
Chromophore Binding Pocket of the Pfr State of Bathy Phytochromes. Photochem. Photobiol..

[75] Kraskov A., Von Sass J., Nguyen A. D., Hoang T. O., Buhrke D., Katz S., Michael N., Kozuch J., Zebger I., Siebert F., Scheerer P., Mroginski M. A., Budisa N., Hildebrandt P. (2021). Local Electric Field Changes during
the Photoconversion of the Bathy Phytochrome Agp2. Biochemistry.

[76] Nguyen A. D., Michael N., Sauthof L., von Sass J., Hoang O. T., Schmidt A., La Greca M., Schlesinger R., Budisa N., Scheerer P., Mroginski M. A., Kraskov A., Hildebrandt P. (2024). Hydrogen Bonding and Noncovalent
Electric Field Effects in the Photoconversion of a Phytochrome. J. Phys. Chem. B.

[77] La
Greca M., Nguyen A. D., Kraskov A., Michael N., Sauthof L., Ebrahim M., Katz S., von Sass J., Hoang O. T., Budisa N. (2025). Propagation of Photoinduced
Electric Field Changes Through Phytochrome and their Impact on Conformational
Transitions. ChemPhyschem.

